# Iodine in Vomiting in Pregnancy

**Published:** 1857-04

**Authors:** 


### MISCELLANEOUS ITEMS.

*Iodine in Vomiting in Pregnancy.* By Dr. EULENBERG.

Dr. J. B. Schmitt some time since recommended the tincture of iodine in the vomiting of pregnant women, when this may be regarded as a neurosis. He gives one case in particular, in which a woman who had, in consequence of vomiting, had four abortions, and became pregnant with her fifth child. Two drops of the tincture given every two hours completely kept the vomiting under, as long as it was continued, and vomiting only returning when it was suspended. At last, a dose two or three times daily, sufficed. Dr. Eulenberg has also met with a remarkable case in a healthy woman, thirty-five years of age, who had suffered from sickness in every pregnancy, and on this occasion had become much reduced. As she was very susceptible to the action of medicines, he ordered her tinct. iod. ℈j.; sp. vini r. ʒiij. Three drops to be taken in water every three hours. By the second day all the most distressing vomiting had disappeared, and she was again able to keep water on her stomach, and in a short time she quite recovered. The *modus operandi* of such small doses of the tincture is not very easily explained; but it is to be observed, that the iodide of potassium exerts scarcely any influence. It is probable that it only influences the vomiting, inasmuch as this is a neurosis, and abnormal condition of the nerves of the stomach.—*Medical Times and Gazette.*

